# Impact of Daily Rhythms and Postprandial Responses on the Plasma Metabolome

**DOI:** 10.3390/ijms27135669

**Published:** 2026-06-23

**Authors:** Tulsi Suchak, Namrata R. Chowdhury, Victoria L. Revell, Cheryl Isherwood, Florence I. Raynaud, Daan R. van der Veen, Nophar Geifman, Debra J. Skene, Matt Spick

**Affiliations:** 1 School of Health Sciences, Faculty of Health and Medical Sciences, University of Surrey, Guildford GU2 7XH, UK; t.suchak@surrey.ac.uk (T.S.); n.geifman@surrey.ac.uk (N.G.); 2 Chronobiology Section, School of Biosciences, Faculty of Health and Medical Sciences, University of Surrey, Guildford GU2 7JG, UK; namrataroy.chowdhury@surrey.ac.uk (N.R.C.); v.revell@surrey.ac.uk (V.L.R.); c.isherwood@surrey.ac.uk (C.I.); d.vanderveen@surrey.ac.uk (D.R.v.d.V.); d.skene@surrey.ac.uk (D.J.S.); 3 Surrey Sleep Research Centre, University of Surrey, Guildford GU2 7XP, UK; 4 Cancer Research UK Cancer Therapeutics Unit, Division of Cancer Therapeutics, The Institute of Cancer Research, London SM2 5NG, UK; florence.raynaud@icr.ac.uk

**Keywords:** metabolomics, chronobiology, postprandial responses, study design, reproducibility, translation

## Abstract

Peripheral blood metabolite concentrations vary with food intake and time of day, risking confounding effects in metabolomics studies with non-standardised sampling conditions or incomplete metadata. Such effects are often overlooked during study design, limiting the clinical translation of biomarkers and wasting resources for researchers, funders and clinicians. In our random sample of 100 human metabolomics studies, 56% did not control for food intake, and 59% did not explicitly control for sampling time. To provide a study design resource, we analysed a liquid-chromatography–mass-spectrometry-targeted dataset from controlled laboratory studies of 24 young, healthy participants (12 male, 12 female) sampled every 2 h for 34 h, with fixed-macronutrient meals provided at set times. Acute postprandial responses were quantified by effect size using pre- and post-meal windows, while daily rhythmicity was assessed using a mixed-effects cosinor model. Analyses were sex-stratified, and metabolites were classified as meal-responsive, time-of-day-responsive, both, or neither. Amino acids and their derivatives showed strong postprandial increases, whereas lipid classes showed minimal changes. Rhythmicity varied across metabolites, enabling the identification of features sensitive to meal timing and/or time of day. These results aim to provide a comprehensive dictionary of metabolite effect sizes for study design and metadata collection to support reproducibility and the clinical translation of potential biomarkers.

## 1. Introduction

Metabolomics is the study of small molecules, metabolic states, biochemical pathways and biomarkers of different conditions [1]. Metabolites act as products or intermediaries in anabolic (building up) and catabolic (breaking down) reactions and vary in concentration in peripheral blood over the 24 h day [2,3,4] due to endogenous biological rhythms and external behavioural or environmental factors such as sleep–wake state and food intake [2,3,4,5]. This sensitivity, however, means that metabolomic measurements are highly susceptible to confounding variables, including variances from meal timing, dietary composition, and time of day [3,6,7,8,9,10]. The latter includes both endogenous circadian rhythm effects and diurnal effects (24-h rhythms driven by external factors and circadian timing).

With regard to postprandial changes, the human metabolome fluctuates as a response, as food intake triggers a series of metabolic processes such as digestion and absorption which induce changes in the levels of circulating metabolites [5,11]. These can resemble or mask physiological signatures, and as a result, studies that do not control for food intake (quantity, composition and timing) may not accurately reflect disease or physiological baseline metabolite levels, but rather the postprandial effects [6,8]. Similarly, metabolism is not static throughout the day, with circadian and diurnal variation controlling many aspects of human physiology [7,12]. Not accounting for time of day can introduce confounding effects that distort statistical associations, especially in observational datasets [7,8]. While it is well documented that both food intake and daily rhythms affect the metabolome, these confounders are not always integrated into metabolomics workflows, especially for large-scale studies where standardisation for sample collection is more limited [13].

These dynamics can pose a serious challenge for biomarker discovery. If sampling is not standardised across a study, a metabolite identified as a disease-associated marker or a pharmacodynamic marker may simply be a proxy for time of day or a postprandial effect. Without controlling for these confounders, findings risk being irreproducible across studies with different sampling techniques [14]. While some metabolomics studies are conducted in the morning following a fasted sleep state, in large-scale studies where blood samples are taken at convenience at single time-points [15], differences in when samples are collected in relation to meals, sleep and circadian phase can create false-positive results [3,4,6], undermining the detection of true metabolic effects.

There are, of course, many other confounders, such as sex, ethnic group, age, smoking status, medication, and comorbidities, to name a few [6,16]. These can be controlled for through either study design or through post-acquisition cohort matching using appropriate metadata. Sampling time is rarely, however, controlled for in ‘omics studies, and whilst fasting is more commonly required, this is far from uniform [13]. For metabolites which are relatively unaffected by meals or by time of day, this may not be an issue. However, for metabolites which are known to be affected by these confounders, the extent to which biomarkers in case-control studies can be robust will depend on whether the effect size is large enough to overcome the confounding variance.

While many studies exist looking at the impact of food consumption or biological rhythms, these have not had a study design or reproducibility focus, which is an important issue for authors, peer reviewers and readers of published research (especially in understanding the limitations of metabolomics studies). In this work, we directly address these issues by analysing a controlled 34-h metabolomics dataset, one comprising 12 males and the other 12 females [3,4], in order to systematically quantify how specific metabolites respond to feeding/fasting and how these responses vary with time of day. By calculating per metabolite changes between pre- and post-meal windows, at different times of day, we aim to generate a reference dataset describing which metabolites are influenced by meal events and which by time-of-day sampling. The resulting metabolite sensitivity dictionary is not intended to provide universal correction factors but, rather, to serve as an assistive resource for study design and interpretation. This is intended to support reproducibility and translation in metabolomics, especially in human studies where dietary behaviour and daily rhythm regulation are often ignored.

## 2. Results

### 2.1. Effect of Meals on Metabolite Concentrations

**Figure 1:**
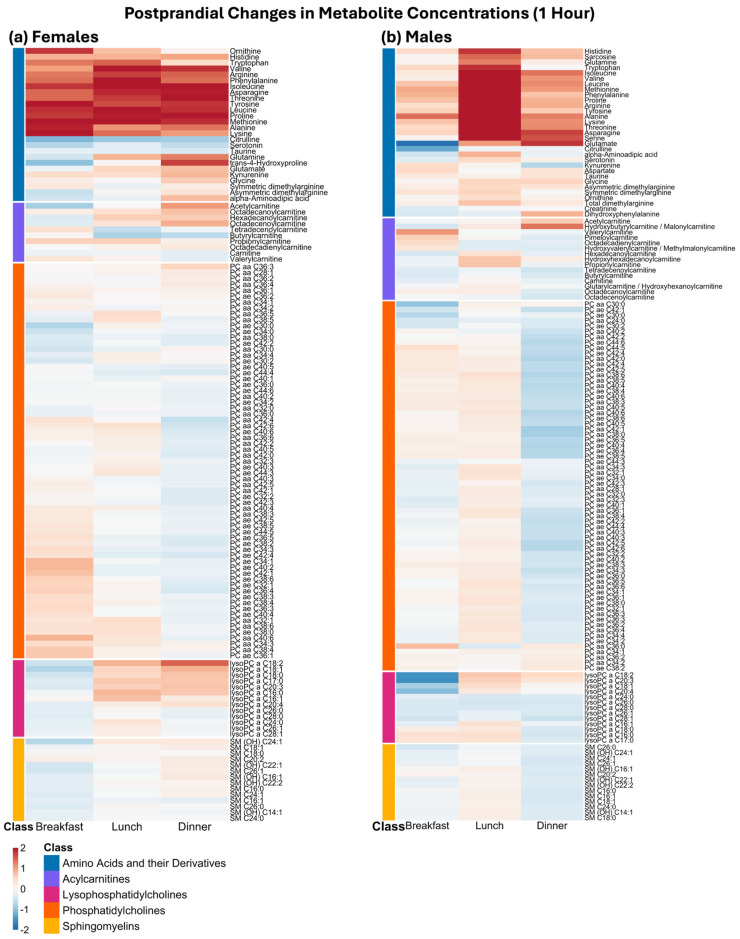
Heatmaps of relative metabolite changes as effect sizes (Cohen’s *d*), representing the difference between preprandial and postprandial concentrations, across meals, stratified by sex, one hour after meal intake. Metabolites are grouped by metabolite class on the *y*-axis and displayed across meal events on the *x*-axis. (**a**) for females, (**b**) for males. Full biochemical names are used for amino acids and their derivatives, and acylcarnitines, while lipid sum-composition annotations are retained for clarity. A complete metabolite-name mapping is provided in the Supplementary Table S1.

Figure 1 shows meal-responsive effect sizes (Cohen’s *d*) for each metabolite, grouped by metabolite class and stratified by sex and meal type one hour after meal intake. Although effect sizes varied across meals, variation was evident following all main meals.

**Figure 2:**
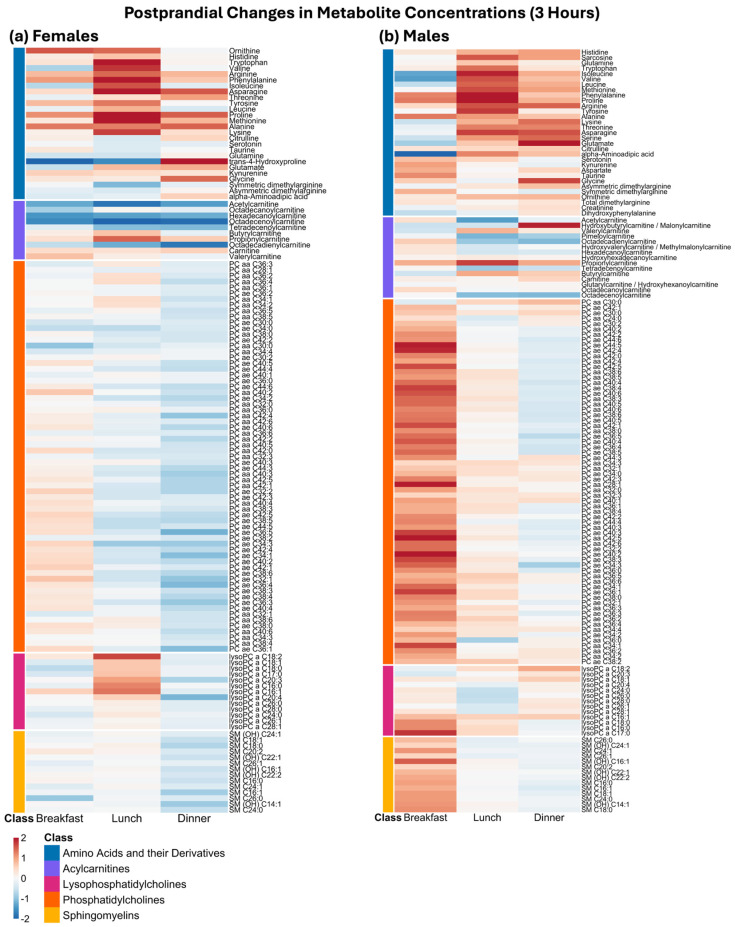
Heatmaps of relative metabolite changes as effect sizes (Cohen’s d), representing the difference between preprandial and postprandial concentrations, across meals, stratified by sex, three hours after meal intake. Metabolites are grouped by metabolite class on the *y*-axis and displayed across meal events on the *x*-axis. (**a**) for females, (**b**) for males. Full biochemical names are used for amino acids and their derivatives, and acylcarnitines, while lipid sum-composition annotations are retained for clarity. A complete metabolite-name mapping is provided in the Supplementary Table S1.

Across breakfast, lunch and dinner, the dominant acute postprandial signature in both females (Figure 1a) and males (Figure 1b) was a strong and consistent increase in amino acids and their derivatives, visible as a broadly positive effect size across this metabolite class. While the overall direction of response was shared between the sexes, the amino acids and their derivatives-associated increase appeared more pronounced in females. While not all metabolites in this class followed the dominant pattern, with some showing weaker or less consistent changes across meals, this finding indicates that within the one-hour post-meal window, amino acids and their derivatives responded rapidly and represent the most sensitive markers of recent food intake. This pattern remained evident at three hours postprandial (Figure 2), continuing to show elevated effect sizes across meals in both sexes.

Lipid-related metabolites exhibited postprandial changes at both one-hour and three-hours after meal ingestion (Figure 1 and Figure 2), with their responses varying by metabolite class, time-point and sex. By contrast to amino acids, most lipid classes showed muted or near-zero changes in effect sizes in the one-hour postprandial window across meals in both sexes. Phosphatidylcholines (PCs), lysophosphatidylcholines (lyso-PCs), and sphingomyelins (SMs) were all largely centred around small effects, indicating a limited acute responsiveness to a meal at this early post-meal time-point. However, at three hours postprandial, SMs and PCs were elevated following breakfast in males, with their response to later meals less consistent. Lyso-PCs and acylcarnitines were altered in both sexes across multiple meals.

### 2.2. Time-of-Day Effects

**Figure 3:**
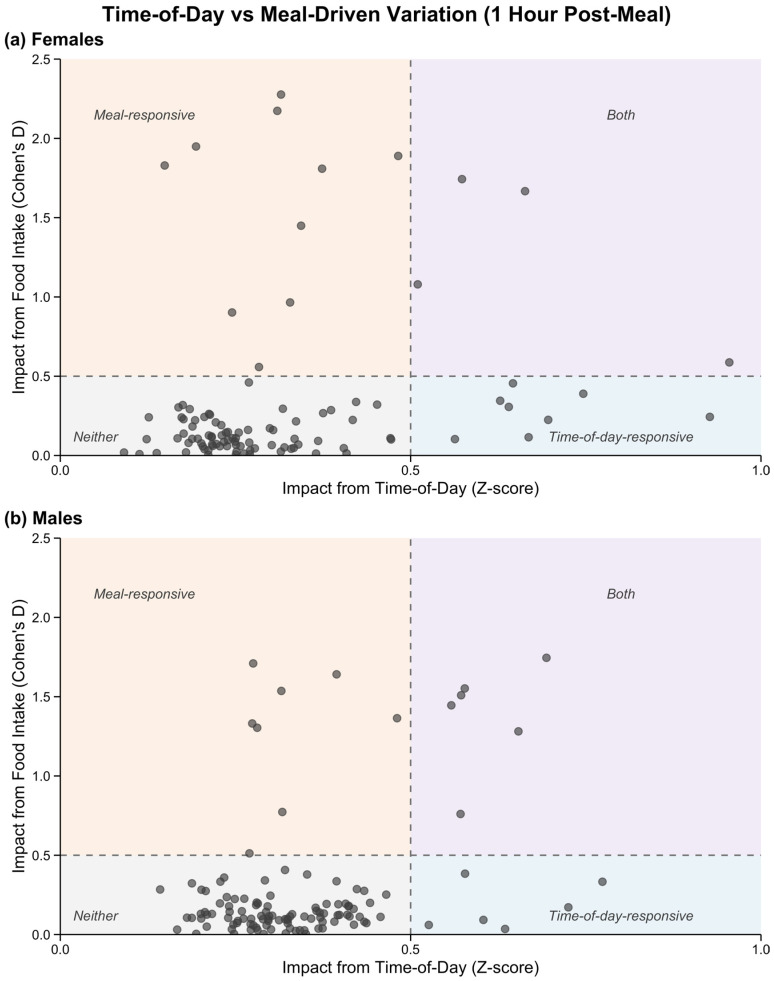
Scatter plot showing the relationship between time-of-day-responsive and one-hour meal-responsive change. The *y*-axis shows the mean postprandial effect size (mean Cohen’s d across meal events), and the *x*-axis shows the rhythmicity z-score derived for metabolites that fit the mixed-effects cosinor model. Dashed lines indicate exploratory standardised effect-magnitude thresholds at 0.5. The resulting quadrants are intended to visualise the relationship between postprandial effect size and rhythmicity magnitude. (**a**) females, (**b**) males. The female and male Cosinor Model Outputs are also reported in Supplementary Tables S2 and S3, respectively.

**Figure 4:**
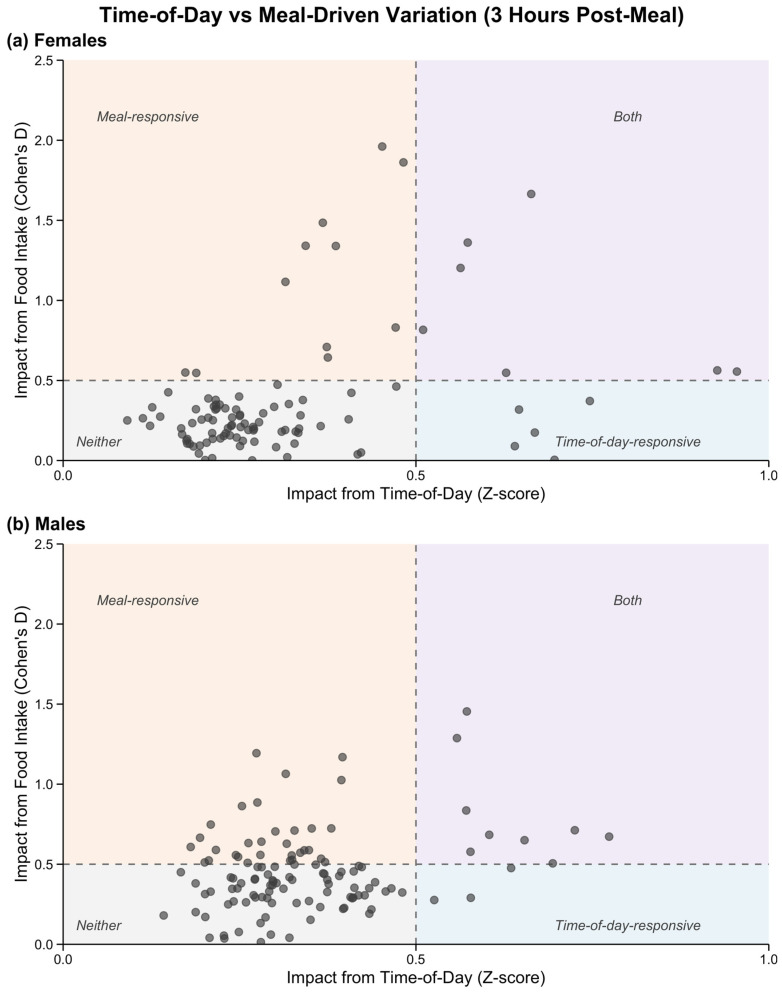
Scatter plot showing the relationship between time-of-day-responsive and three hour meal-responsive change. The *y*-axis shows the mean postprandial effect size (mean Cohen’s d across meal events), and the *x*-axis shows the rhythmicity z-score derived for metabolites that fit the mixed-effects cosinor model. Dashed lines indicate exploratory standardised effect-magnitude thresholds at 0.5. The resulting quadrants are intended to visualise the relationship between postprandial effect size and rhythmicity magnitude. (**a**) females, (**b**) males. The female and male Cosinor Model Outputs are also reported in Supplementary Tables S2 and S3, respectively.

As can be seen in Figure 3 and Figure 4, across both sexes, metabolites spanned a wide range of time-of-day-responsive effect sizes. A heuristic threshold corresponding to moderate standardised effect magnitude (|effect size| ≥ 0.5) was used pragmatically to show metabolites as meal- or time-of-day-responsive. Several of the metabolites with meal-responsive changes did not show time-of-day-responsive effect sizes (females: *n* = 10, *n* = 11, males: *n* = 8, *n* = 31, at one and three hours, respectively). A distinct subset of metabolites showed time-of-day-responsive effect sizes alongside minimal meal-responsive change (females: *n* = 8, *n* = 5, males: *n* = 6, *n* = 3, at one and three hours, respectively). A smaller group of metabolites exhibited both large meal effect sizes and high time-of-day-responsive effects (females: *n* = 4, *n* = 7, males: *n* = 6, *n* = 9, at one and three hours, respectively).

Metabolites were also stratified by effect size magnitude for both meal-responsive and time-of-day-responsive analyses (Table 1). The meal-responsive metabolites were assigned according to the larger |Cohen’s d| value observed at either one hour or three hours postprandial, whereas time-of-day-responsive metabolites were assigned according to their z-score, with metabolites lacking a valid cosinor model fit, for females, *n* = 30 (23.1%), and for males, *n* = 23 (16.3%), excluded from this analysis.

Overall, the metabolites were distributed across all effect sizes when considering meal intake influence, with a greater proportion in the small and moderate effect size categories for males, and in the small effect size for females (Table 1). Both groups had metabolites in the large effect size category, with 16.9% (*n* = 22) in females and 12.1% (*n* = 17) in males.

As seen in Table 1, time-of-day-responsive metabolites also spanned a range of effect sizes, although their distribution differed from that observed for meal-responsive effects. For both groups, the greatest proportion of metabolites belonged to the small effect size category, with 68% (*n* = 68) in females, and 84.7% (*n* = 100) in males, while few to none had large effect sizes with 2% (*n* = 2), and 0% (*n* = 0), for females and males, respectively. Although only effect sizes ≥ 0.5 were considered indicative of meaningful meal- or time-of-day-responsive effects, metabolites within the small effect size range (0.2 ≤ small effect size < 0.5) may still exhibit weak responses and should not be interpreted as entirely unaffected.

### 2.3. Metabolite Sensitivity Dictionary

To translate these analyses into a form that supports improved study design and interpretation, metabolites were assigned into one of the four categories based on the combined evidence from the meal responses and 24 h cosinor modelling: (i) meal intake, (ii) time of day, (iii) both, or (iv) neither. A metabolite was classed as meal-responsive if either the one hour or three hours postprandial Cohen’s *d* effect size was ≥0.5. Metabolites were classed as time-of-day-responsive if they exhibited a significant cosinor model fit after Benjamini–Yekutieli false discovery rate correction, with non-significant or non-fitting metabolites classified as not exhibiting detectable time-of-day effects.

In females, 48.5% were classified as time-of-day-responsive only, with a further 13.1% classified as both meal- and time-of-day-responsive. In males, 45.4% of metabolites were classified as time-of-day-responsive only, with an additional 24.1% classified as meal- and time-of-day-responsive. Taken together, a large proportion of metabolites in both sexes were sensitive to time-of-day variation, and a substantial proportion were influenced by both meal intake and time of day.

A full classification table for all metabolites is provided in Supplementary Materials, Table S4 for females, and Supplementary Table S5 for males. In addition, a sensitivity analysis of the meal-responsive classifications using |Cohen’s d| thresholds of 0.2, 0.5 and 0.8, corresponding to small, moderate and large effect sizes, is presented in Supplementary Tables S6 and S7 for females and males, respectively. The 0.5 threshold was used as the primary threshold here for the meal-response classification because it represented a conventional moderate effect and provided a pragmatic balance between the broad inclusion produced by the 0.2 threshold and the more restrictive 0.8 threshold. Furthermore, it should be noted that the z-score thresholds were included only to illustrate variation in rhythm magnitude and were not used to redefine time-of-day responsiveness in the primary classification tables.

### 2.4. Assessment of Gaps in the Literature

Across all the studies, single-time-point sampling was the most common design, with relatively few incorporating repeated sampling (Table 2).

Reporting of sample collection time was more variable, with only a small proportion of studies (11%) having reported exact sampling times, while others (15%) described broader time-of-day windows (e.g., ‘morning’). A subset also used relative timing, most commonly in relation to clinical events or controlled intervention studies. However, a substantial proportion of the studies (59%) had not reported any information on sampling time. This lack of reporting was observed across both observational and clinical study designs.

Fasting status was also inconsistently reported. While 44% of studies explicitly stated that samples were collected under fasting conditions, often following an overnight fast, 56% did not report fasting status at all. When clinical event-driven and controlled intervention studies were excluded from consideration, 52% of the remaining studies still did not use fasting protocols.

**Figure 5:**
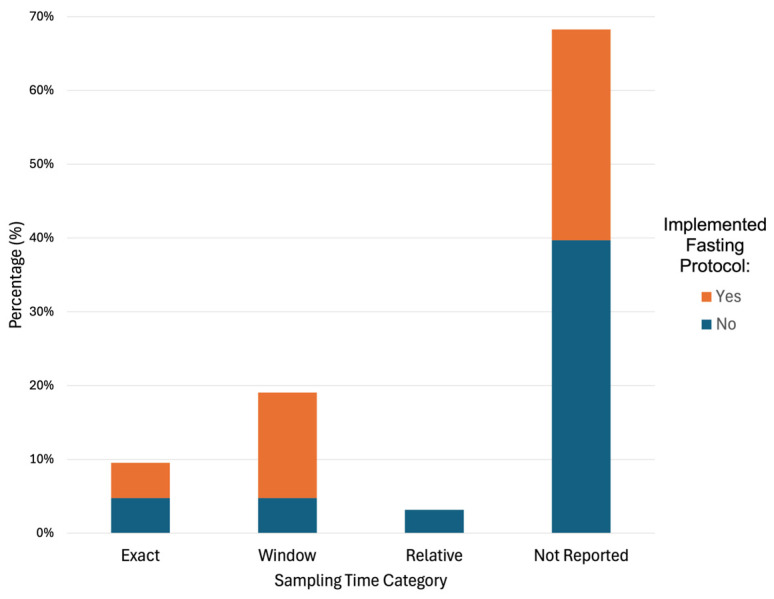
Fasting status across sampling time reporting categories in observational studies. Stacked bar chart showing the proportion of studies classified by sampling time reporting category (exact, window, relative, or not reported) and fasting status (fasted vs. non-fasted) within observational studies.

This pattern was further illustrated when restricted to observational studies, where fasting status remained inconsistently reported across sampling time categories (Figure 5). Notably, among studies that did not report any sampling time information, fasting protocols were not consistently applied, indicating that missing metadata is not limited to a single variable but reflects broader gaps in study design and reporting.

## 3. Discussion

Although the sensitivity of the metabolome to feeding and time of day is well established [3,4,9,10,17,18], in practice, information on meal proximity and sampling time is not usually incorporated into metabolomics study design or analysis pipelines. Across our sample of metabolomics studies, 59% did not report any form of sampling time, and fasting protocols were not applied in 56% of cases. Together, these findings suggest a disconnect between known biological drivers of metabolite variation and how studies are designed and reported.

A key implication for biomarker discovery is the misinterpretation of metabolite responses when sampling occurs under unrecorded feeding/fasting conditions or at single time-points [19,20]. Amino acids and their derivatives exhibited moderate to large meal-responsive effect sizes across sexes, indicating that these features respond quickly and substantially to food ingestion, with their acute postprandial change consistent with the previous literature [21,22]. In addition to this, the magnitude of these responses differed between sexes, consistent with broader evidence in sex-specific metabolomic variation [23], and remained detectable several hours after meal intake, demonstrating both sex-specific and temporal effects. Moreover, responses were not uniform within this metabolite class, with some of the amino acids and their derivatives (e.g., glycine) showing weaker signals and highlighting that there are metabolite-specific responses even within the classes.

This finding presents a reproducibility challenge because amino acids and their derivatives are frequently proposed as candidate biomarkers [24,25,26]. A simple search of the PubMed database for “amino acid” and “biomarker” reveals 7927 publications [27] between 2020 and 2025. Based on our sample, this is suggestive that several thousand studies did not implement a consistent fasting protocol and/or did not record the sampling time. Many of these studies will also then be included in systematic reviews or meta-analyses, allowing poor study design to be propagated through the literature. Under convenience sampling, an observed difference in an amino acid could be reflecting differences in time since the last meal [21] rather than a disease effect. Without explicit control or adjustment for meal timing, meal-responsive signals or residual noise will, therefore, risk being misinterpreted as disease-associated effects [17,21,28], inflating false-positive findings and reducing reproducibility.

Although some variation in effect magnitude was observed across feeding events, suggesting that meal characteristics (e.g., composition) and daily rhythms influence how strongly the metabolome changes after eating, metabolomic differences were evident following all major feeding events. While this is useful in controlled laboratory studies, designed to measure nutrient responses, it is a greater issue for epidemiological studies, where sampling time and time since last meal are not usually standardised or recorded [13,15]. In those settings, systematic differences in sampling relative to recent food intake between participant groups could generate metabolomics associations reflecting this variability rather than the exposure or disease of interest, reinforcing the need to account for meal timing and time-of-day variation in study design and analysis.

The 24 h rhythm analysis, using cosinor fitting, also emphasises that timing effects in metabolomics are not just related to acute meal responses [2]. By combining the cosinor-based rhythmicity analysis with the mean postprandial effect sizes, we show that metabolites differ in whether they are primarily influenced by feeding, by time of day, by both, or by neither. Metabolites that show strong time-of-day effects but minimal meal responsiveness may be biased mainly by the time of sampling, while metabolites the large postprandial effects but weak time-of-day effects are more sensitive to time since last meal. For metabolites that exhibit strong postprandial and time-of-day effects, convenience sampling is most likely to generate confounding unless both meal and time of day are explicitly accounted for.

These results are directly relevant to large epidemiological resources that collect non-fasted samples [15,29,30]. Non-fasted sampling may be informative for testing whether biomarkers perform under real-world conditions; however, such datasets are only interpretable if key metadata are provided to be able to separate feeding/meal effects from other sources of biological variation, else misleading associations and poor replication become more likely [14,20,31]. In effect, this becomes a ‘risk of bias’ in meta-analysis of biomarkers, which unfortunately is often not explicitly considered. By recording metadata related to meal intake and time of sample collection, we can improve translation of metabolomics data into clinical settings by reducing confounding effects.

Stronger minimum standards for sampling and reporting are, therefore, needed in metabolomics research. Where feasible, studies should record the sampling time, the time since the last caloric intake, and basic information about recent intake (e.g., meal type and approximate macronutrient content). These steps would reduce meal- and time-of-day-responsive confounding, improve reproducibility and provide a more robust basis for 24 h rhythm modelling. Existing standards, such as the Metabolomics Standards Initiative [32], have rightly been concerned with instrument protocols and feature identification, but good study design must also take account of biological variation and its responses to meals, sleep and sampling time.

A number of weaknesses, and areas for further work, should also be considered. First, the Biocrates system used here is targeted; while this improves standardisation, accuracy, and reproducibility [33], it does restrict the number of features covered compared to untargeted profiling and may bias the results towards metabolite classes that are well represented in targeted panels [33,34]. Future work could extend these analyses using broader untargeted platforms to capture a wider range of metabolites. Second, the dataset utilised here was collected in a controlled but small cohort of healthy, young, adult participants, and the findings may not generalise to older or paediatric populations or to clinical cohorts with differing dietary patterns or biological rhythms [35,36,37], or indeed may be influenced by batch effects, reagent lots, instrument drift, and other technical factors that may differ between studies. In addition, because meal timing could not be completely separated from circadian rhythm influences, the stronger morning postprandial responses seen within our results may in part also reflect circadian or sleep–wake influences alongside direct meal effects. Consequently, the findings should be considered exploratory, given the small n (especially so for the sex-stratification analyses), and the metabolite sensitivity dictionary should be interpreted as a reference resource rather than as a universal correction tool. Third, the potential confounding effects of pharmacological exposures could not be modelled in this study, as all participants were medication-free, except for oral contraceptives in the female group. Commonly used medications can produce measurable changes in the plasma metabolome; for example, paracetamol has been reported to affect lipid-related metabolic pathways [38] and could introduce bias into metabolomic associations if not recorded or adjusted for. Fourth, our sample of metabolomics datasets to determine how often these effects were controlled for was selected from a prior work analysing open-access publications, as open-access journals differ from paywalled journals in impact, author demographics, and methodological rigour.

In conclusion, by both separately and jointly quantifying postprandial effects and time-of-day rhythmicity, we aimed to provide a practical, metabolite-level guide for study design and interpretation, highlighting metabolites that could potentially be confounded under convenience sampling versus those that may be less sensitive to meals and clock timing and therefore more robust candidates for biomarker discovery.

## 4. Materials and Methods

### 4.1. Study Design and Sampling

**Figure 6:**
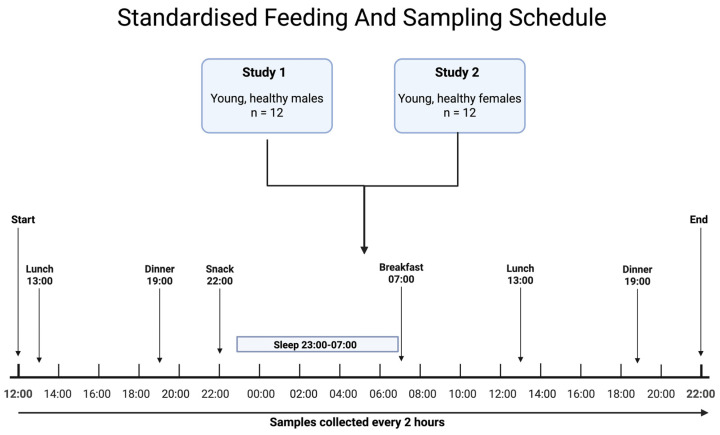
Study design and sampling protocol. Two separate controlled studies were conducted in healthy participants: one in males (*n* = 12) and one in females (*n* = 12), giving a total of 24 participants across both studies. Samples were collected every 2 h for 34 h from 12:00 on Day 1 to 22:00 on Day 2, resulting in 18 time-points per participant. Participants remained under controlled laboratory conditions with standardised environmental light, sleep, meals and posture, with designated periods for freely moving daytime activity. Meals were provided at standardised times: breakfast (07:00), lunch (13:00), dinner (19:00), and a late-evening snack (22:00).

The data utilised is from two independent controlled studies conducted with 12 male participants in one study and 12 female participants in the second. All participants were considered young and healthy. Full details of their demographics, inclusion and exclusion criteria have been previously published [3,4]. Samples were collected across 34 h, every two hours from 12:00 on Day One to 22:00 on Day Two, with 18 time-points in total. The participants followed a regular sleep/wake schedule (23:00–07:00). Meals were provided at standardised times: breakfast (07:00), lunch (13:00), dinner (19:00), and a late-evening snack (22:00). This protocol is shown in Figure 6. Meals had a fixed-macronutrient composition, designed to reflect the UK dietary guidelines (targeted at fat 35%, carbohydrate 50%, protein 15% of energy), with limited variations across the meal types (see Supplementary Materials, Table S8, for additional information on specific meal breakdowns). All meals were the same across both days. Water was available ad libitum throughout the protocol.

### 4.2. Targeted Metabolomics Measurement

Plasma metabolite concentrations were measured using the AbsoluteIDQ^®^ p180 targeted metabolomics kit (Biocrates Life Sciences, Innsbruck, Austria) using a previously described method [39,40]. In brief, plasma samples (10 µL) were prepared according to the manufacturer’s instructions, adding several stable isotope-labelled standards prior to the derivatisation and extraction steps. Metabolites were measured on a Xevo TQ-S triple quadrupole mass spectrometer (MS) coupled to a Waters ACQUITY ultra-performance liquid chromatography (UPLC) system with a Waters BEH C18 reversed-phase 1.7 µm, 2.1 × 75 mm column (Waters, Milford, MA, USA). Using either liquid chromatography–tandem mass spectrometry (LC–MS/MS) or flow injection analysis–tandem mass spectrometry (FIA–MS/MS), up to 184 metabolites from five metabolite classes can be quantified: acylcarnitines, amino acids, biogenic amines, glycerophospholipids (PCs, lyso-PCs) and sphingomyelins. Amino acids and biogenic amines were measured by LC-MS/MS; acylcarnitines, PCs, lyso-PCs, and SMs were measured by FIA–MS/MS.

Amino acids and biogenic amines (LC-MS/MS) were quantified against seven-point calibration curves (Cal1–Cal7), using external calibrators alongside isotope-labelled internal standards. For the FIA-MS/MS measurements, a subset of acylcarnitines (C0, C2, C3, C4, C5, C6, C8, C10, C12, C14, C16, C18) was quantitative and calibrated via linear regression against authentic standards. The remaining acylcarnitines, glycerophospholipids and sphingolipids were classed as relative-quantitative measurements, as each one was referenced against a single representative isotope-labelled internal standard per class, without a multi-point calibration curve. Most lipid species, therefore, represented summed concentrations of isobaric and isomeric species sharing the same total carbon number and double bond count.

Samples were randomised, and three quality control (QC) levels of lyophilised human plasma were included per 96-well plate. Intra- and inter-plate normalisation was performed using four replicates of QC level 2 (QC2) and Biocrates MetIDQ software (version as per source studies [3,4]). Metabolites were excluded if >25% of values were below the limit of detection (LOD), outside the quantification limits, blank out of range or the QC2 coefficient of variation exceeded 30% [4,40].

### 4.3. Sex-Stratified Analysis

All statistical analyses were conducted separately for males and females. Sex was treated as a potential confounder for both the baseline metabolite levels and response patterns. This stratification was used to avoid potentially pooling heterogeneous dynamics. Results are reported in parallel for males and females to highlight any similarities and differences in postprandial and rhythmic patterns.

### 4.4. Meal Windows and Quantifying Postprandial Responses

To quantify acute postprandial food response effects, pre- and post-meal windows were defined as being relative to each scheduled meal. For each meal event, the pre-meal window corresponded to the sample collected approximately one hour before the meal, while the post-meal window corresponded to the sample collected approximately one hour and three hours after the meal. As participants had two instances of lunch and dinner across the study period, the mean was used.

For each metabolite, participant and meal event, we computed a postprandial effect size using Cohen’s *d* [41] to prioritise the metabolites with consistent responses. This was calculated as:

$$Cohen^{’}s\;d=\frac{\mu_{pre-meal}-\mu_{post-meal}}{\sigma },$$

where $\mu_{1}$ and $\mu_{2}$ are the group means, and σ is the pooled standard deviation. For the interpretation of the Cohen’s *d* values, absolute values were categorised into thresholds: |d| < 0.2 (negligible), 0.2 ≤ |d| < 0.5 (small), 0.5 ≤ |d| < 0.8 (moderate), and |d| ≥ 0.8 (large) [41]. These categories were used for descriptive summaries of effect size distributions. For classification purposes, metabolites with |d| ≥ 0.5 were considered meal-responsive. This threshold was selected as a pragmatic criterion corresponding to a conventionally defined moderate effect size, allowing potentially meaningful responses to be prioritised while limiting classification based on very small effects. It was not intended to represent a definitive biological boundary.

### 4.5. Assessing Rhythmicity with Mixed-Effects Cosinor Modelling

To identify the rhythmic patterns in metabolite levels across the day-night cycle, cosinor analysis was performed in MATLAB (version R2025b, MathWorks Inc., Natick, MA, USA), with a previously described linear mixed-effect cosinor model [39] determining daily rhythmicity. In brief, two cosinor models with a level and sloping mesor were fitted to raw metabolite concentrations (μM) using the linear mixed-effects models and accounting for repeated measures, and individual variability as a random effect. Significant models (*p* < 0.05) were then identified and used to estimate peak time, amplitude, and mesor levels. Given the high number of metabolites tested, *p*-values for these metabolites were then adjusted using Benjamini–Yekutieli false discovery rate correction [42], given the correlated and interdependent nature of metabolite concentrations [19]. Metabolites were classed as exhibiting time-of-day effects based on statistically significant cosine fit using adjusted *p*-values, and those without a significant fit or those with an amplitude confidence range that included 0 were classed as not exhibiting detectable time-of-day effects. To quantify time-of-day variation, for metabolites with a cosinor model fit, each metabolite was characterised by a z-score derived from the model. This was calculated as:

(1) $$Z-score=\frac{x-\mu }{\sigma },$$

where x is the observed value of the metabolite concentration at peak time (acrophase), *μ* is the mean concentration for the metabolite, and *σ* represents the standard deviation of a metabolite’s concentration.

A z-score represents the deviation of an individual value from the mean, while Cohen’s *d* represents the difference between two means relative to their variability [43].

Although Cohen’s *d* and z-scores quantify different statistical properties, both express differences in units of standard deviation (pooled standard deviation in the case of Cohen’s *d*). Here, the thresholds were used jointly and pragmatically as heuristic indicators of effect magnitude to support effect-magnitude summaries and visualisation rather than to imply formal statistical equivalence between the two measures. Accordingly, a threshold of |effect size| ≥ 0.5 was selected as an interpretable criterion for identifying metabolites with moderate or larger responses.

### 4.6. Classifying Metabolites by Source of Variation

Metabolites were then organised according to whether the observed variability was primarily explained by (i) meal intake, (ii) time of day, (iii) both, or (iv) neither. This categorisation was based on the combined evidence from the postprandial response analyses and rhythmicity modelling. Metabolites were labelled as meal-responsive when they showed a postprandial effect (|Cohen’s *d*| ≥ 0.5) in the meal-response analysis, and time-of-day-responsive based on the presence of a significant cosinor model fit following multiple testing correction. This ensured that all metabolites could be consistently classified, including those for which z-score effect size calculation was not possible. Metabolites not meeting either criterion were classified as neither, representing relatively stable features, and were classified as both when both criteria were met. To assess the sensitivity to the selected meal-response cutoff at|Cohen’s d| ≥ 0.5, the classification was repeated using |Cohen’s d| thresholds of 0.2, 0.5 and 0.8, corresponding to conventional small, moderate and large effect sizes. The definition of time-of-day responsiveness remained unchanged and continued to require a significant cosinor fit after false discovery rate correction. For those rhythmic metabolites, the same thresholds were also applied to the absolute z-score as an exploratory summary of rhythm magnitude, although these z-score thresholds were not used to alter the primary time-of-day classification.

### 4.7. Literature Assessment of the Unmet Need

Finally, 100 randomly sampled studies from a previously described dataset of metabolomics papers were reviewed to assess gaps in study design relating to food intake and biological rhythms in the Open Science Framework (OSF) [44]. This approach was used to assess current gaps in study design. The source dataset comprised open-access, English language metabolomics articles indexed in PubMed, PubMed Central and the Directory of Open Access Journals (via OpenAlex), published between 2013 and 2024. Database-specific searches used metabolomics-related terms, with the complete search strings provided in Supplementary Materials, Table S9 and as detailed in our previously published protocol and work [45].

The available source dataset formed a convenience sampling frame of open-access metabolomics publications, and records were randomly selected and screened until 100 studies meeting the additional inclusion criterion of analysing human biofluids had been identified. Reviews, methods papers and studies that did not analyse human biofluids were excluded. The included studies were then reviewed to assess the gaps in study design relating to food intake and biological rhythms. The full literature assessment is available in Supplementary Materials, Table S10.

For each study, information was recorded on biofluid type, sampling context, sampling design, reporting of sample collection time and implementation of a fasting protocol. Sampling context was classified as clinical, observational or controlled trial. Clinical sampling referred to acute or event-driven settings (e.g., emergency presentation), observational sampling included routine or scheduled collections not linked to an acute clinical event (this included biobank-based studies), and controlled trials involved structured experimental interventions with predefined sampling protocols.

Sample collection timing was classified as ‘Exact’ when an explicit clock time was reported, ‘Window’ when a broader time-of-day interval was provided, ‘Relative’ when sampling time was reported only in relation to an event or intervention, and ‘Not reported’ when no information was provided. ‘Implemented fasting protocol’ indicated that a clearly defined and consistent fasting procedure was applied across all relevant participants. Studies with unclear, mixed, or inconsistently applied fasting conditions were classified as not implementing a consistent fasting protocol. Results were tabulated in order to identify the gaps in study design in the literature.

## Figures and Tables

**Table 1:** Distribution of metabolites across effect-magnitude bins stratified by sex. Metabolites were grouped based on absolute effect size. Meal-responsive effects were defined using the larger |Cohen’s d| value at 1 h or 3 h postprandial, and time-of-day-responsive effects using z-scores. Metabolites without a valid cosinor fit were not included in this analysis. Effect sizes were categorised as: (negligible effect) < 0.2; 0.2 ≤ (small effect size) < 0.5; 0.5 ≤ (moderate effect size) < 0.8; 0.8 ≤ (large effect size). Metabolites were considered to have a meaningful effect if either effect size was ≥ 0.5.

Sex	Classification	Number of Metabolites	Negligible (*n*)	Small (*n*)	Moderate (*n*)	Large (*n*)
Female	Meal-responsive	130	42	57	9	22
	Time-of-day- responsive	100	20	68	10	2
Male	Meal-responsive	141	10	83	31	17
	Time-of-day- responsive	118	6	100	12	0

Individual cosinor model fits are provided in Supplementary Materials, Tables S2 and S3.

**Table 2:** Sampling practices stratified by study context (*n* = 100). Studies where a fasting protocol was present, but not implemented for all participants, were considered to be ‘not implemented’.

Category	Subcategory	Observational (*n*)	Clinical (*n*)	Controlled Trial (*n*)
Sampling Design	Single time-point	51	12	2
	Repeated	10	5	12
	Time-series	1	0	5
	24 h-pooled sample	1	1	0
Sampling Time	Exact	6	0	5
	Window	12	3	0
	Relative	2	6	7
	Not reported	43	9	7
Implemented Fasting Protocol	Implemented	30	3	11
	Not implemented	33	15	8

## Data Availability

The datasets analysed within this study were generated and previously described by Davies et al., 2014 [3], and Honma et al., 2019 [4]. Data access conditions remain as described in those publications. Data derived during this study are provided in the Supplementary Materials and additionally at the Zenodo repository with https://doi.org/10.5281/zenodo.20699015.

## Supplementary Materials

The following supporting information can be downloaded at: https://www.mdpi.com/article/10.3390/ijms27135669/s1.

## Abbreviations

The following abbreviations are used in this manuscript:

PCs	Phosphatidylcholines
Lyso-PCs	Lysophosphatidylcholines
SMs	Sphingomyelins
MS	Mass spectrometer
UPLC	Ultra-performance liquid chromatography
LC-MS/MS	Liquid chromatography–tandem mass spectrometry
FIA–MS/MS	Flow injection analysis–tandem mass spectrometry
QC	Quality control
QC2	Quality control level 2
LOD	Limit of detection
OSF	Open Science Framework
